# *In vivo* Observation of Tree Drought Response with Low-Field NMR and Neutron Imaging

**DOI:** 10.3389/fpls.2016.00564

**Published:** 2016-05-06

**Authors:** Michael W. Malone, Jacob Yoder, James F. Hunter, Michelle A. Espy, Lee T. Dickman, Ron O. Nelson, Sven C. Vogel, Henrik J. Sandin, Sanna Sevanto

**Affiliations:** Los Alamos National LaboratoryLos Alamos, NM, USA

**Keywords:** water, NMR, tree, health, flow, neutron imaging, drought, hydraulics

## Abstract

Using a simple low-field NMR system, we monitored water content in a living tree in a greenhouse over 2 months. By continuously running the system, we observed changes in tree water content on a scale of half an hour. The data showed a diurnal change in water content consistent both with previous NMR and biological observations. Neutron imaging experiments show that our NMR signal is primarily due to water being rapidly transported through the plant, and not to other sources of hydrogen, such as water in cytoplasm, or water in cell walls. After accounting for the role of temperature in the observed NMR signal, we demonstrate a change in the diurnal signal behavior due to simulated drought conditions for the tree. These results illustrate the utility of our system to perform noninvasive measurements of tree water content outside of a temperature controlled environment.

## 1. Introduction

Changes in tree water status, i.e., the availability of water to the tree, are an indication of hydraulic performance. Long-term changes are related to environmental stresses such as drought (Tyree and Sperry, 1989; Sevanto et al., 2005; Zweifel et al., 2005), or to pathogen or insect activity (Paine et al., 1997; Umebayashi et al., 2011), whereas diurnal changes are associated with a plant's ability to mitigate short term water deficit using stored water (Sevanto et al., 2005; Hölttä et al., 2009; Vergeynst et al., 2015). There are many ways to measure tree water status, though few are low-cost, nondestructive, and scalable. Existing nondestructive methods such as observing stem diameter variation (Daudet et al., 2005), make assumptions about cavitation and elastic capacitance of the stem (Perämäki et al., 2001; Hölttä et al., 2002, 2012), while others, such as gamma ray observations (Edwards and Jarvis, 1983), require expensive equipment. More commonly, changes in the water status of trees are observed by measuring leaf or xylem water potential, however this is a destructive measurement, and one that does not lend itself to rapid, large scale observations. Neutron imaging allows for quantitative observation of water transportation through plants as shown here and by Defraeye et al. (2014). Its use is currently limited to small plants and it requires the use of contrast agents which may interfere with plant biology. However, the time and distance scales of the measurement are excellent. Techniques using nuclear magnetic resonance (NMR) have also been pursued (Van As et al., 1994; Rokitta et al., 2000; Windt et al., 2006, 2009, 2011; De Schepper et al., 2012; Jones et al., 2012; Windt and Blümler, 2013), since it is non-destructive, sensitive to the protons in water, i.e., the hydrogen nuclei, and can be run continuously for months, as shown here.

Previous work observing tree water status using NMR has included MRI machines (Rokitta et al., 2000; Windt et al., 2006, 2009; Van As, 2007; Kimura et al., 2011; De Schepper et al., 2012; Van As and van Duynhoven, 2013), which allow detailed imaging of water location, but are constrained by tree size. Permanent magnet systems have also been demonstrated (Windt et al., 2011; Jones et al., 2012; Windt and Blümler, 2013), but their large size and mass creates a challenge for building a network of detectors on many trees, or for working on plants with diameters greater than 1 cm. Here we provide a detailed analysis of the data obtained with a previously reported (Yoder et al., 2014) simplified low field NMR system, weighing just a few kilograms. Such low field measurements are made using static magnetic fields below 10 mT, in contrast to fields of several Tesla used in traditional NMR and MRI machines. While the lower field reduces the signal strength, our system was able to measure the water status of a tree in a greenhouse for 2 months under simulated drought and other extreme conditions. To understand the physical source of the NMR signal we acquired NMR data simultaneously with neutron imaging measurements on branches. By observing uptake of heavy water by the branches with both systems, we show that the NMR signal is primarily due to water being transported through the plant. We also show how the NMR signal can detect the bulk loss of water in a living tree, and by carefully accounting for the role of temperature, we show how the diurnal signal behavior varies as the tree enters drought conditions. While previous studies (Windt et al., 2011; Jones et al., 2012) have addressed the role of temperature on magnet stability, they have not explicitly accounted for the role of temperature in the NMR signal and the detector. Our method isolates the variation in the signal due to biological changes from that due to temperature, providing a low-cost, scalable, and noninvasive method for determining the water status of mature trees over long periods of time.

## 2. Methods and materials

### 2.1. NMR introduction and equipment

**Figure 1:**
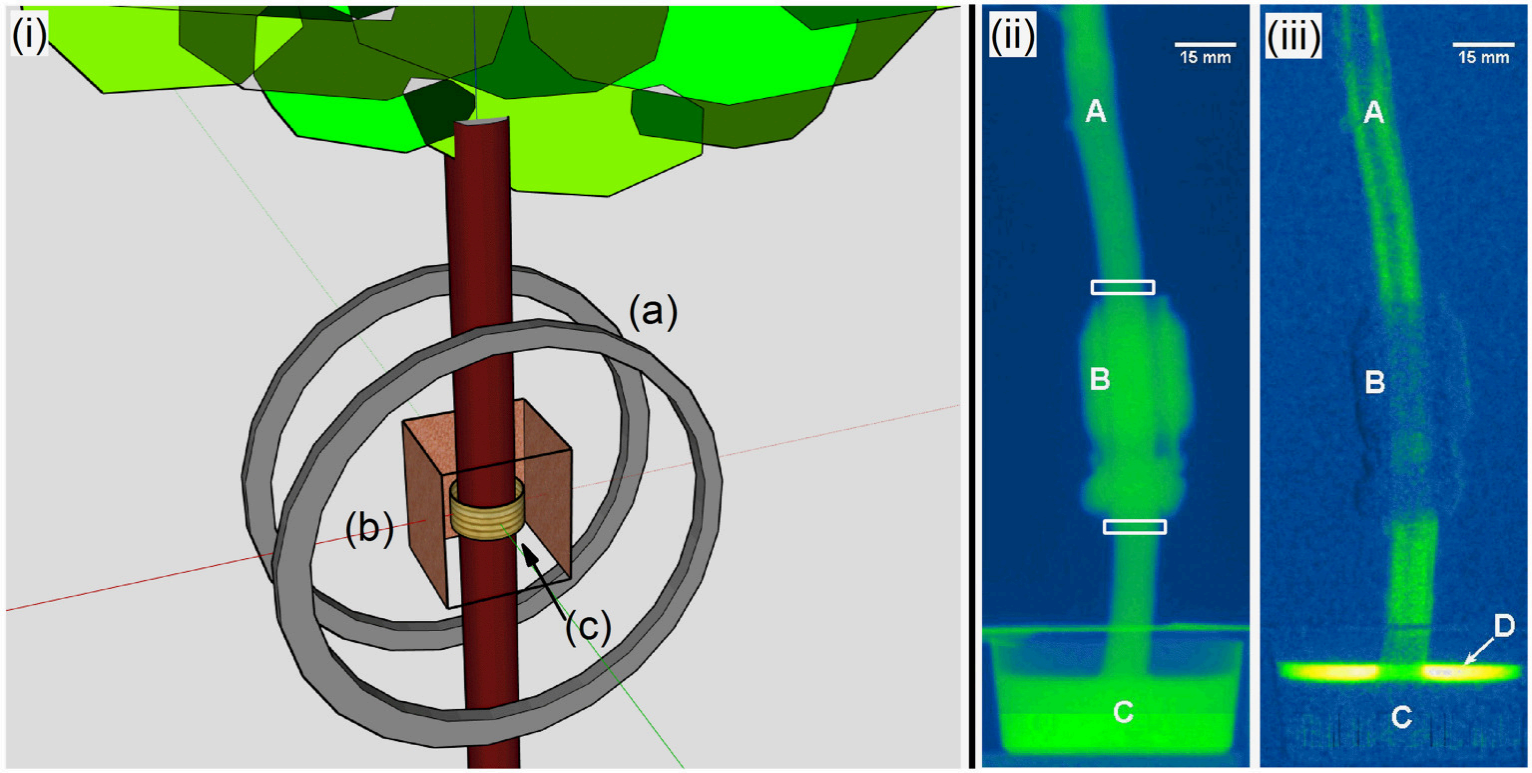
**(i)** A schematic of our low field NMR system used to measure the water content in a tree shows (a) the 41 cm diameter Helmholtz coils which produce ${\vec{B}}_{0}$; (b) copper shielding, with one panel removed for clarity, used to block rf noise; and (c) the solenoid, wrapped around a former on the tree's trunk, which both produces ${\vec{B}}_{1}$ and detects the resulting NMR signal. **(ii)** A representative neutron image of a branch showing (A) the stem; (B) the NMR solenoid; (C) the cup holding the heavy water; and the regions of interest as highlighted by the white boxed. **(iii)** Subtracting subsequent neutron images from the first creates difference images. Such images clearly show the uptake of the heavy water through the branch, even revealing a section of non-conductive heartwood running down the center of this branch. Difference images also help reveal any physical movement of the system, and provide a direct measurement of the amount of water taken up by the plant (D).

In NMR, a population of target nuclei, with spin greater than zero and gyromagnetic ratio γ, are polarized with a static magnetic field ${\vec{B}}_{0}=B_{0}ẑ$ (Abragam, 1961). A resonant radio frequency (rf) pulse, with frequency *f*_*NMR*_ = γ · *B*_0_/2π is then applied to the nuclei. The nuclei respond by producing an oscillating magnetic field, with frequency *f*_*NMR*_, that can be detected with an AC magnetometer. The experimental data shown in this paper were obtained using a custom NMR system (Figure 1i) described in detail in Yoder et al. (2014). In brief, the system consists of a set of Helmholtz coils to produce ${\vec{B}}_{0}$ with a magnitude of 860 μ*T*. Typical NMR experiments are performed at much higher ${\vec{B}}_{0}$ fields, with state of the art MRI systems operating at several Tesla. The higher field leads to more polarization and a larger *f*_*NMR*_, which makes signal detection easier. However such high field systems are complicated and expensive, and not suitable for field work. By working at a lower field, we sacrifice the signal obtained from a single measurement, but can provide a simple, inexpensive, and rugged system. Since a tree is an inherently static object with relatively slow dynamics, unlike a patient in an MRI machine, we can still obtain an excellent signal to noise ratio (SNR) by averaging many measurements.

Using a current limited power supply (PWS4205, Tektronix, Beaverton, OR, USA), the stability of our ${\vec{B}}_{0}$ was observed through variation in *f*_*NMR*_, which stayed within 37 Hz of 36,600 Hz for 2 months. To minimize any heating effects of the coil, a fan was continuously used to blow air across the system. A solenoid, itself part of a resonant circuit with a quality factor of 35, was wrapped around a coil former that fit around the tree's 3.5 cm diameter trunk. This coil produced both the rf magnetic field ${\vec{B}}_{1}$ used to excite the hydrogen nuclei, and served as the NMR signal detector.

NMR signals were acquired using a free induction decay (FID) pulse sequence, which consists of a single optimal 90° excitation pulse of 270 μs for the aspen data and 40 μs for the juniper and pinon data followed by signal acquisition. Signal was acquired every millisecond from 5 to 12 ms after the pulse for the aspen data and from 3 to 25.6 ms for the juniper and pinon data. The difference in the two acquisition schemes was due to improvements in the experimental protocol. To obtain an adequate SNR, each datapoint is the average of the FFT of 1500 FIDs taken over 30 min for the aspen data, 3400 FIDs taken over 17 min for the juniper data, and 5000 FIDs taken over 25 min for the pinon data, with the peak being the observed NMR signal. In our Aspen experiments, the observed NMR signal was found to return to thermal equilibrium governed by an exponential time constant *T*_1_ of 200 ms.

#### 2.1.1. NMR temperature correction

The average daily temperature variation in the greenhouse was 10 K, with an average daily high near 305 K. When left unaccounted for, these temperature variations alone produce a significant variation in the observed NMR signal. For the temperature range of our experiments, our NMR signal *S* has a known temperature dependency (Abragam, 1961) of

(1) $$\begin{array}{l} S\propto \frac{W_{c}}{kT}, \end{array}$$

where the water content *W*_*c*_ is a function of the relative density of the water producing the signal within the coil, *k* is Boltzmann's constant, and *T* is sample temperature in Kelvin. In order to accurately measure the variation in the observed NMR signal due to biological changes in the tree, we needed to remove this known temperature variation from our observed signal.

In order to remove additional systematic effects due to temperature, it was necessary to characterize the temperature sensitivity of our system. To do this a second solenoid, rigidly mounted near the detector of our NMR system, was connected to a function generator to provide a constant AC signal that was observed with our detector. By tracking the variation in this test signal over 1 week in the greenhouse, we found that our detector had a linear temperature dependency, probably due to variation in the resistance of the detector circuitry. This simple test was very reproducible and can easily be configured for any similar system. We stress that the temperature dependency we report is particular to the system we used. Additional detector circuits we tested varied considerably in their response to temperature. Incorporating our observed temperature dependence with Equation 1 lead to an expression we refer to as the temperature model:

(2) $$\begin{array}{l} S\propto \frac{W_{c}}{kT}\cdot \left[1-0.0013\left(T-275\right)\right], \end{array}$$

where the factor of [1 − 0.0013(*T* − 275)], obtained using a least-squares fit of the test signal to the greenhouse temperature, measured in the vicinity of the NMR system, accounts for the particular linear dependence of our detector circuitry on temperature. This model predicted variation in the NMR signal due solely to temperature. To remove the now known variation due to temperature effects from *S* and directly measure the water content we simple divide *S* by [1 − 0.0013(*T* − 275)]/*T*. The resulting signal variation should be due entirely to variation in the water content of the tree and not temperature effects. We note that the precise physical source of the variation in the water content is unknown. It can be attributed not only to variation in the amount of water inside the NMR coil but also to changes in the distribution of water within the stem, and the decay rate *T*_2_ of the NMR signal.

### 2.2. Long-term NMR aspen treatment

The long-term NMR experiments were performed in a greenhouse on a potted, 5 m tall aspen tree (*Populustremuloides*), native to New Mexico, from July 11, 2013 until September 5, 2013. The potted tree was regularly weighed using a digital scale (HD150S, Sartorium AG, Gottingen, Germany) to assess water consumption. Sensors adjacent to the tree were used to measure relative humidity (CS215, Campbell Scientific, Logan, UT, USA), photosynthetically active photon flux density (PPFD) (LI-190 Quantum sensor, Li-Cor Inc., Lincoln, NE, USA), and atmospheric temperature (CS215, Campbell Scientific, Logan, UT, USA), with the measurements recorded to a datalogger (CR23X, Campbell Scientific, Logan, UT, USA) every five minutes. The tree was manipulated to encourage water loss in several ways. Watering of the tree was stopped after the first 8 days. On day 25 the crown was removed 1 m above the NMR solenoid and the cut sealed with parafilm. On day 39 a second unsealed cut was made 10 cm above the detector coil. On day 43, toward the end of the experiment, the tree was girdled (continuous strips of phloem and bark removed) above and below the detector coil to accelerate drying.

### 2.3. Simultaneous NMR and neutron imaging

#### 2.3.1. Neutron imaging introduction and equipment

To verify the physical source of the hydrogen nuclei that contribute to the NMR signal of our system, neutron imaging experiments were performed simultaneously with low field NMR measurements on branches of juniper (*Juniperus monosperma*) and pinon pine (*Pinus edulis*), chosen because of their significance to NM forests and their variation to drought tolerance Dickman et al. (2015). These experiments were performed at the Lujan Center, a part of the Los Alamos Neutron Science Center (LANSCE, http://lansce.lanl.gov/lujan/index.shtml), using the thermal neutrons at Flight Path 05. The neutron detector was a PaxScan 2520 (Varian Medical Systems, Salt Lake City, UT, USA) detector custom modified for neutron imaging. The NMR experiments were performed using the system described above, but with a smaller solenoid to accommodate the roughly 1 cm diameter branches.

#### 2.3.2. Branch treatment and experimental protocol

The branches, with dimensions in Table 1, were taken roughly 24 h before an experiment began from mature trees growing in ambient conditions with no stress treatment. The cuttings were placed in water and left in the dark to induce stomatal closure and minimize biological activity. Within an hour of starting an experiment, 1 cm of bark and phloem were removed from the bottom of the branch and the newly exposed surfaces sealed with PTFE tape to ensure water was taken up through the xylem. The branches were then exposed to photosynthetically active radiation from a 12 W LED growth lamp (AgroLED, Spain). The light intensity was verified using a handheld quantum meter (LightScout, Spectrum Technologies, Inc.m Aurora IL, USA) and had a maximum value of 850 μmol/m^2^/s at the top of the branch. The light induced stomatal opening was verified using a leaf porometer (SC-1 Leaf Porometer, Decacon Inc., Allyn, WA). When the branches and equipment were ready, the ends of the branches were placed into cups of 99.9% deuterated water and simultaneous recording of the NMR and neutron data began. Atmospheric temperature and relative humidity (CS215, Campbell Scientific, Logan, UT, USA) in the measurement space were recorded (CR23X, Campbell Scientific, Logan, UT, USA) every five minutes. At the end of the experiments the branches were harvested for leaf area measurements (Leaf area meter LI-3100C, Li-Cor Inc. Lincoln, NE, USA). The stem diameter, both with and without bark, was measured along two orthogonal axes at the location of the NMR coil to determine branch total, xylem, and the combined phloem and bark cross-sectional areas.

**Table 1:** **Characteristics for the branches used in the simultaneous NMR and neutron imaging measurements**.

**Sample**	**Mean branch cross-section (mm^2^)**	**Mean xylem cross-section (mm^2^)**	**Phloem and bark cross-section (mm^2^)**	**Leaf area (cm^2^)**
Pinon	123 (±2)	63 (±1)	60 (±1)	777.0 (±0.2)
Juniper	93 (±1)	65 (±1)	27 (±1)	996.4 (±0.2)

*The cross-sections were calculated assuming a circular branch cross-section. The representative diameter of each branch was obtained by averaging three pairs of measurements, taken along orthogonal axes, within the volume observed by the NMR coil*.

The toxic effects of deuterated water on mammals has been reported (Czajka et al., 1961), but less is known about its effects on plants. To ensure that the branches were behaving normally while fed the heavy water, stomatal conductance measurements were made before and after experiments to verify that the branch was transpiring. Continuous measurements were logistically infeasible due to the combination of radiation hazards and rf noise that the device produced which interfered with the NMR signal. With the juniper branch, we replaced the heavy water with regular water 2.5 h into the experiment to verify that saturation of the NMR signal was due to saturation of the branch by deuterium and not to a heavy water mediated slow down of plant activity. This was not done on the pinon branch because of the limited time available in the neutron beam. The obtained neutron radiographs were corrected for background noise of the camera (dark current correction), intensity fluctuations of the incident beam, spatial inhomogeneities in the beam and detector (flat field correction) and scattering of neutrons by the setup (black body correction) as described in Defraeye et al. (2014). After these corrections, the original neutron intensity data was converted to obtain the neutron attenuation/mm. The final images were analyzed using ImageJ to assess the change in neutron attenuation for two regions of interest. These were 3 mm tall windows that spanned the width of the branch and were made just above and below the NMR coil (Figure 1ii). Flow rates were calculated from the time lag in the appearance of heavy water in the neutron signal above and below the NMR coil (Table 2). The time lag was determined as the time shift giving the highest correlation between the neutron signals measured above and below the NMR coil.

**Table 2:** **Signal change rates in %/h, with the standard error in parentheses, for the regions of interest above and below the NMR coil were calculated assuming a linear time dependency**.

	**Juniper**				**Pinon**	
	**Stage 1**	**Stage 2**	**Stage 3**	**Stage 4**	**Stage 1**	**Stage 2**
Above	-26.1 (±1.4)	−2.2 (±0.1)	30.2 (±1.5)	2.9 (±0.4)	−1.3 (±0.1)	−0.6 (±0.1)
Below	−23.3 (±1.3)	−2.7 (±0.1)	35.1 (±2.6)	1.7 (±0.1)	−1.8 (±0.1)	−0.7 (±0.1)

*The corresponding rates for the same branch are, for four of the six measurements, statistically different. This discrepancy is likely due to the roughness of the linear approximation as a measurement tool as much as it is due to biological variation in the regions of interest. By correlating the signal variation flow rates for the juniper were found to be 9.2 (±1.5) cm/h and 12.2 (±0.5) cm/h*.

## 3. Results

### 3.1. Long-term NMR aspen measurement

**Figure 2:**
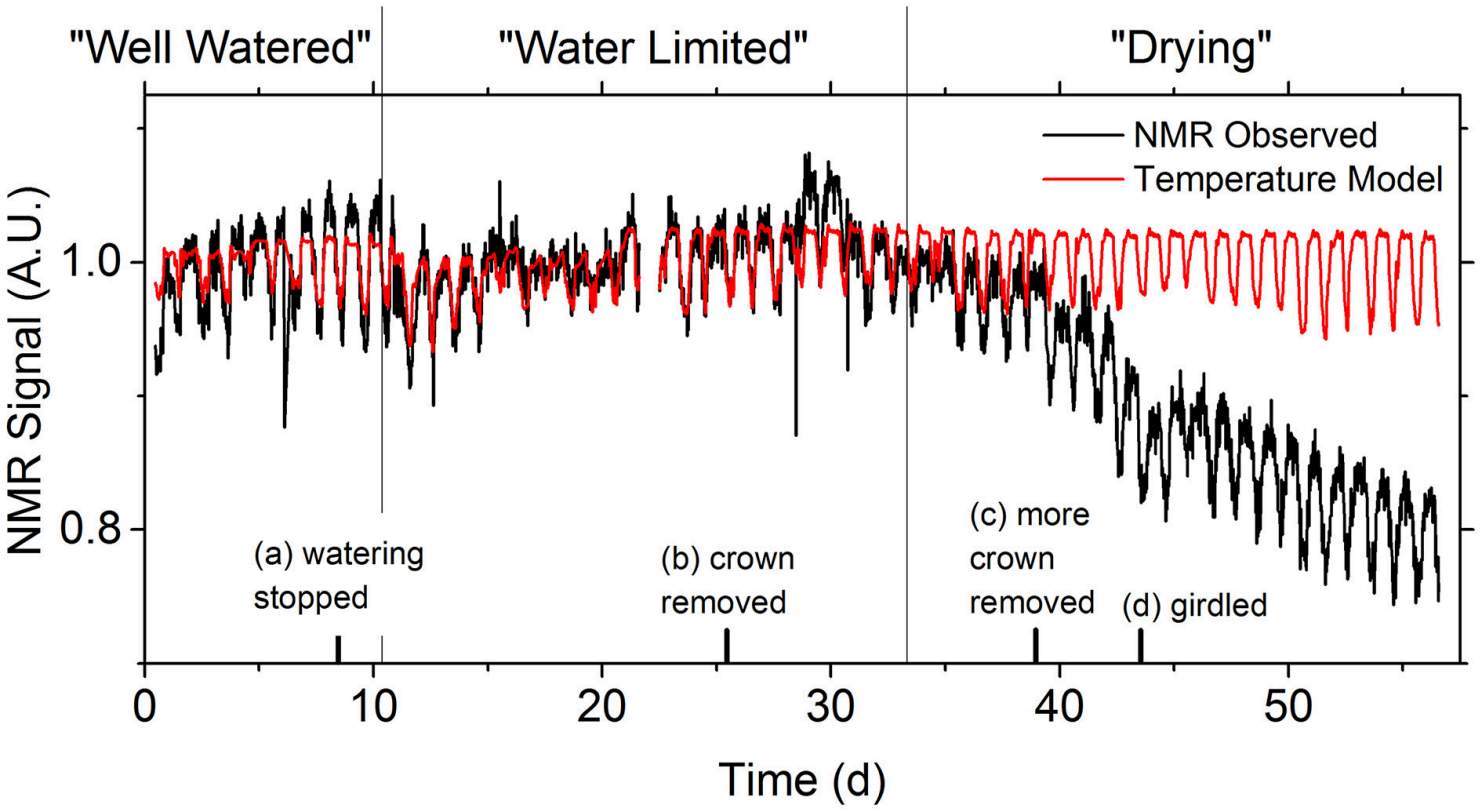
**The diurnal variation in the NMR signal (black line) is apparent for the entire 2 months of data acquisition**. The temperature model (red line) of Equation 2 visually accounts for the vast majority of the signal variation during the water limited epoch, but not for the well watered or drying epochs, since the water content was not constant. To encourage water loss, the tree was manipulated in several ways: (a) watering was stopped; (b) the crown was removed 1 m above the detector coil, with the cut sealed with an impermeable barrier; (c) the crown was removed 10 cm from the detector coil and the cut left unsealed; (d) strips of phloem were removed above and below the detector coil to cease carbohydrate transport. Epochs were determined based on NMR signal response to the manipulations.

In the long-term NMR experiment in the greenhouse, the NMR signal observed from the aspen tree had a clear diurnal pattern across the entire experiment, with the signal varying within 3% of its daily mean value (Figure 2). Similar diurnal oscillations in water content have been reported before with NMR, (Windt et al., 2011; Jones et al., 2012) however these reports did not fully account for the possible role of temperature in the observed signal variation. We note that the general trend in this diurnal oscillation is for a stronger signal during the night than during the day, which is consistent both with the expected biological behavior of trees (i.e., a higher water content at night when evaporative demand is low) and the temperature model of Equation 1.

The well watered pattern continued for 2 days after watering was stopped. Once the tree entered the water limited stage the bulk flow of water through the tree ceased. This was confirmed by measuring the change in mass of the potted tree. During this epoch the NMR signal variation was mostly due to variation in temperature. This continued for over 20 days, including 8 days after the top of the tree had been removed (Figure 2), before the downward trend in the NMR signal indicated the tree had entered its drying stage.

**Figure 3:**
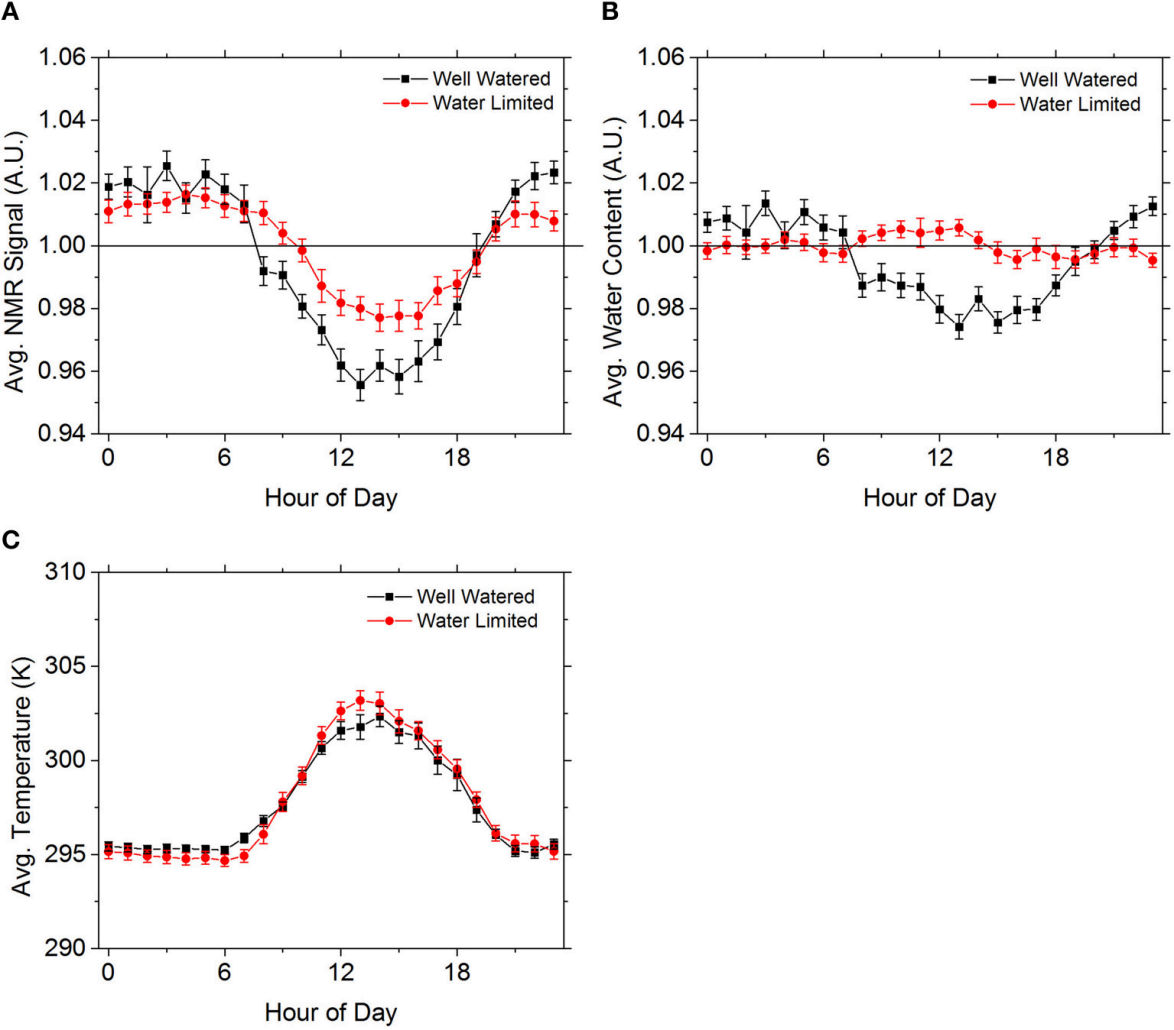
**The average NMR signal by hour (A) for the well watered (black squares) and water limited (red circles) epochs are qualitatively distinguishable**. While the trend is very similar, the well watered signal is stronger at night and weaker during the day compared to the water limited data. By removing the signal variation predicted with the temperature model of Equation 2 **(C)** the differences in the signal behavior become clearer. The water limited data is well accounted for using the temperature model, but this is not the case for the well watered data. The average daily temperatures **(B)** between the two epochs are quite similar. Error bars come from the standard error.

Focusing on the well watered and water limited epochs, their respective average diurnal NMR signals are distinguishable, with the variation in the well watered signal being larger than the corresponding water limited data despite the average daily temperature variations being nearly identical (Figures 3A,B). The behavior of the water content between the two epochs, however, is quite different (Figure 3C). For the well watered tree removing the variation predicted by the temperature model alone fails to account for a large amount of the NMR signal variation. The signal is stronger than expected at night, and weaker than expected by temperature alone during the day. This suggests that a biological influence, such as transpiration, varies the water content. In contrast, for the water limited tree, the signal variation is almost entirely accounted for with the temperature systematics alone, implying no biological influence is changing the water content.

**Figure 4:**
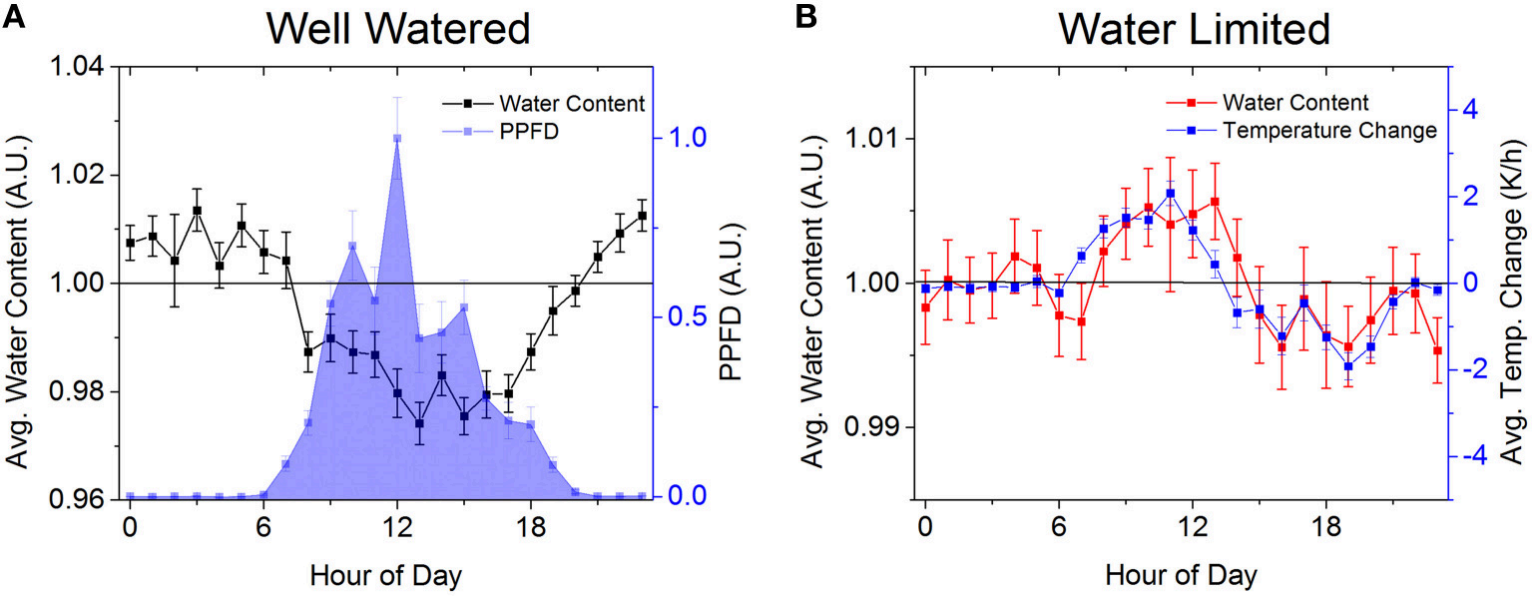
**The gross behavior in the well watered tree's average hourly water content (black squares) (A) is well correlated with environmental variables such as air temperature (*r* = −0.93, *N* = 24, *p* = 5e–11), relative humidity (*r* = 0.89, *N* = 24, *p* = 9e–9) and PPFD (*r* = −0.77, *N* = 24, *p* = 1e–5, blue squares), showing that the variation in water content is due to the tree responding to its surroundings**. In contrast, the small variation in the water content (red squares) for the water limited tree **(B)** is well correlated with the average hourly temperature changes (blue squares, *r* = 0.78, *N* = 24, *p* = 9e-6) at the detector, since rapid changes in temperature produce a thermal gradient across the sample coil and limit the accuracy of the temperature model. Error bars come from the standard error.

Potential explanations for the diurnal variation in water content include behaviors triggered by light, and thermal diffusion effects (Figure 4). For the well watered tree the change between the tree having a surplus and deficit of water content is consisten with diurnal changes in light availability and evaporative demand. The variation is also comparable to the amplitude of elastic volume variations observed in field conditions for Scots pine (*PinussylvestricL*.) (Sevanto et al., 2002) and beech (*FagussylvaticaL*.) (Sevanto et al., 2003). While the variation in the water content for the water limited data is quite small, there is a pattern that temperature alone apparently cannot explain. However, by looking at the average rate of change of temperature in the greenhouse, the data suggests that rapid temperature changes make determining the temperature of the water inside the tree difficult, with the observed variation in water content consistent with a temperature inaccuracy of just a few Kelvin. The small variation is encouraging because, while wood is a poor heat conductor (Glass and Zelinka, 2010), we do not see a very large temperature difference between the trunk and the surroundings for trees of this size. However the residual's presence implies that, for larger trees, temperature may become a bigger problem and methods for measuring trunk temperature as a function of position may be necessary.

### 3.2. Simultaneous NMR and neutron imaging

**Figure 5:**
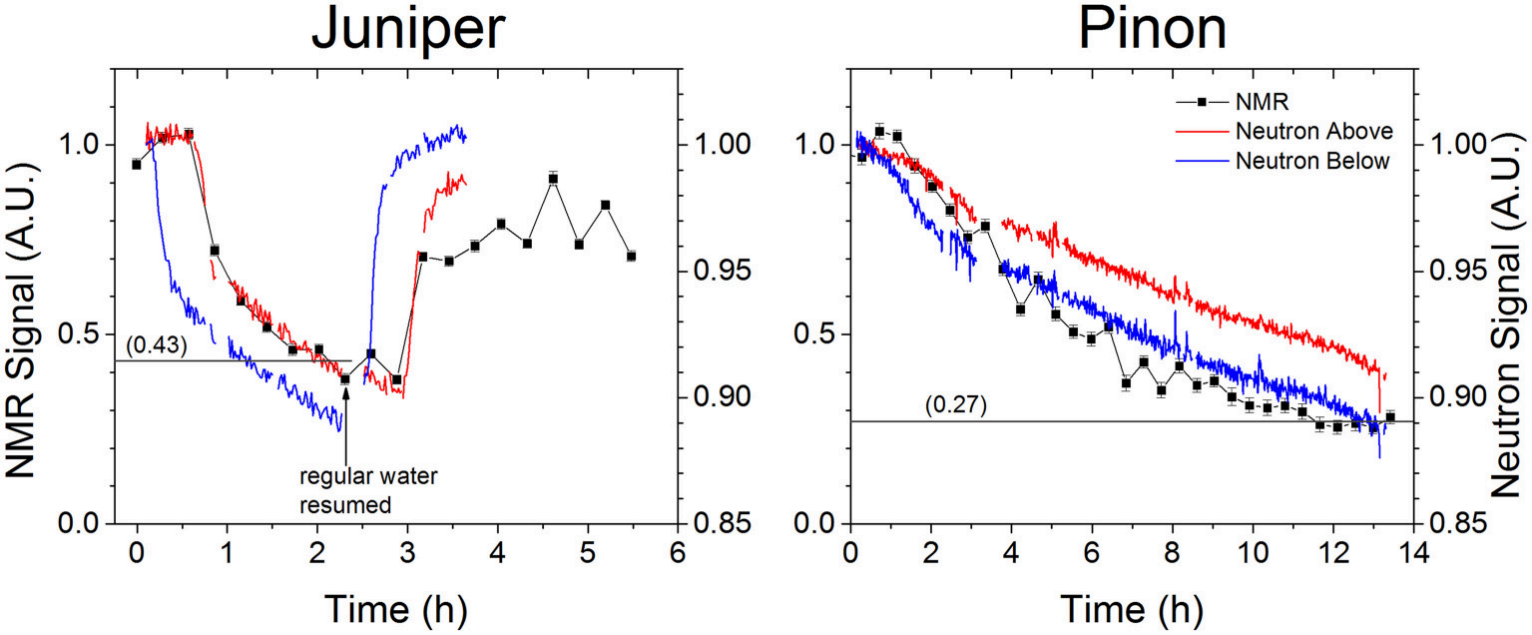
**The raw NMR signal (black squares) and neutron signals measured for volumes above (blue lines) and below (red lines) the NMR detector coil (see Figure 1ii for regions of interest) for both juniper and pinon branches decrease as the deuterated water replaces regular water through the branch**. Heavy water was added to the branch at 0 h while regular water was resumed at 2.5 h for the juniper branch. The NMR signals converged to 43 and 27% of their initial values for juniper and pinon, respectively, demonstrating that the majority of the NMR signal is due to water being rapidly transported through the plant. The relatively higher converged value for the juniper may have been limited by the presence of heartwood that was not transporting water (Figure 1iii). The difference in time scales for the two species reflects their respective uptake rates, as given in Table 2, and the duration of the data acquisition.

In the combined NMR neutron imaging experiments, both the NMR and neutron signals showed a relatively rapid initial decline followed by a second slower phase as the branches took up and became saturated with heavy water (Figure 5). For both branches, the raw NMR signal declined over 50%, variation far too large to explain with temperature alone, while the decline in the neutron signal intensity was less than 20%. The direct observations of deuterated water uptake with neutron imaging verify that the change in NMR signal was not due to the branch simply drying out. The recovery of the signals on the juniper branch after resuming regular watering confirmed that the branch stayed functional throughout the experiment (Figure 5).

The signal decline due to heavy water uptake was faster in juniper than in pinon. In juniper a constant reduced NMR signal intensity was obtained in approximately 2 h while in pinon it took 12 h to see convergence of the NMR signal. For both pinon and juniper, the neutron signal did not converge to a constant intensity within our experimental time. This is because the neutron signal is able to measure the entire sample volume with equal sensitivity compared to the NMR coil, which is more sensitive to water transported through the xylem, as discussed below. The slow signal saturation in pinon could not be entirely accounted for by differences in flow rates. Flow velocity in pinon was 9.7 cm/h and in juniper 12.2 cm/g, which can be entirely explained by the difference in leaf area (Table 1). The pinon and juniper branches were also similar in xylem diameter and conductive xylem area (Table 1), but the pinon branch had much thicker bark, increasing its total cross-section, and the combined phloem and bark area by 25% and 50% relative to juniper. The slow saturation of the NMR and neutron signals could therefore be attributed to a greater influence of the phloem and bark tissues to the signal in pinon than in juniper. The phloem and bark tissues exchange water with the xylem, and would eventually be saturated with heavy water as well, but the hydraulic conductance between the xylem and phloem and bark is several orders of magnitude lower than the axial conductivity in the xylem (Sevanto et al., 2011), and consequently saturation is very slow. The neutron signal strength is directly proportional to the sample thickness, and therefore if larger phloem and bark thickness explained the differences in saturation time entirely, we would expect pinon to take twice as long as juniper. However the factor of six increase in saturation time observed with the NMR signal in pinon compared to juniper indicates that the hydraulic conductivity between the xylem and phloem and bark must also differ between these species.

**Figure 6:**
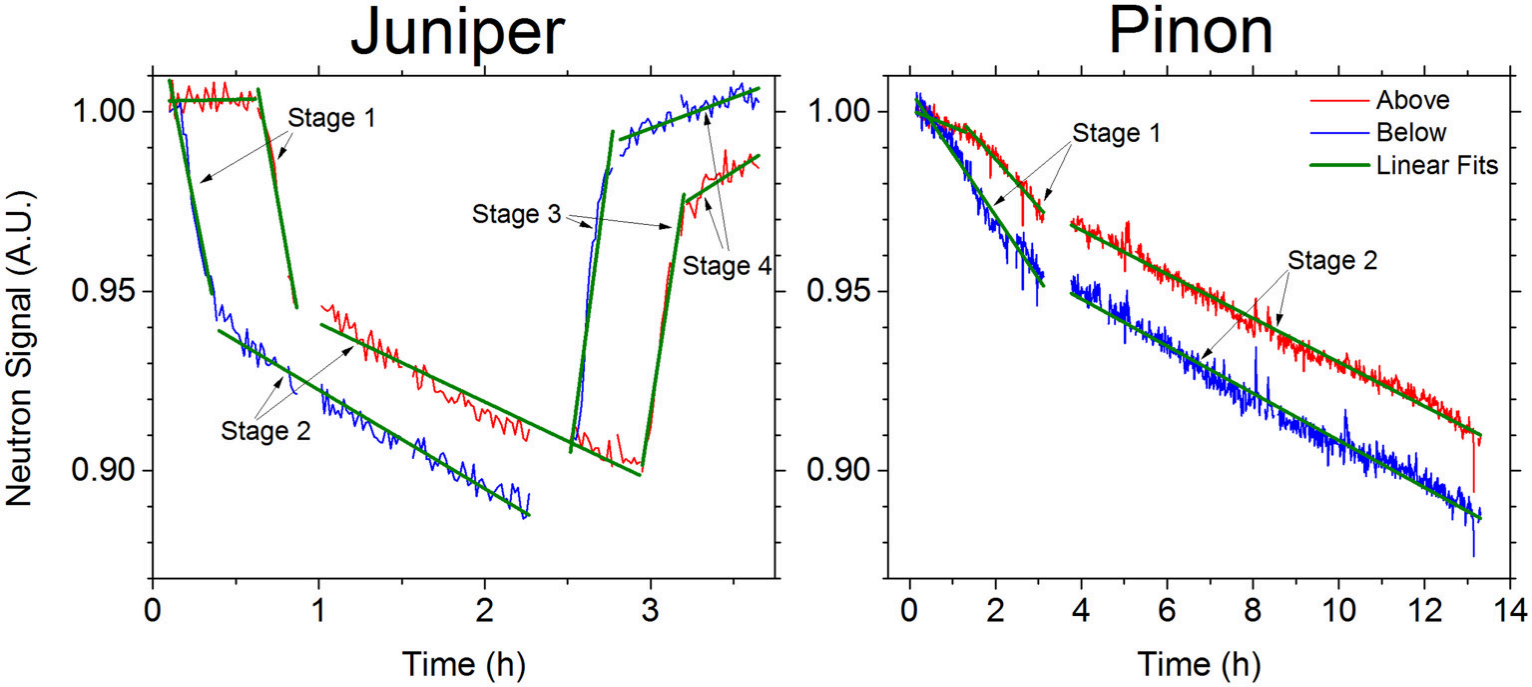
**The neutron signals above (blue lines) and below (red lines) the NMR detector coil (see Figure 1ii for regions of interest) for the juniper and pinion branches showing the regions corresponding to the linear fits (green lines) in Table 2**. The difference in time scales for the two species reflects their respective uptake rates, as given in Table 2, and the duration of the relevant data acquisition.

Focusing on the neutron data, we can seperate the response of the branches into distinct phases (Figure 6). In pinon the decline rates observed in Stage 1, lasting for 150 min, for the regions of interest were nearly double the decline rates of Stage 2 (Figure 6, Table 2). In juniper the Stage 1 rate was roughly ten times the Stage 2 rate and lasted for 14 min (Figure 6, Table 2). When normal watering resumed after the NMR signal saturated, the magnitude of the signal recovery rates in Stage 3 were greater than the Stage 1 rates, before resuming a similar recovery rate in Stage 4 (Figure 6, Table 2). Previous observations on leaves suggest that the relative “fast” and “slow” uptake phases may result from two distinct compartments contributing to the signal at different proportions and saturating at different intensities (Cruiziat et al., 1980; Zwieniecki et al., 2007). The first compartment, consisting of the conductive xylem, rapidly saturates with water and contributes most to the initial “fast” phase. After the first compartment is filled, filling of the second compartment, mostly phloem and bark with some contribution of living cells in the xylem then dominates the response. The rate of flow to the second compartment affects the observed fast and slow rates. In pinon, the flow to the second compartment appears strong enough to reduce the difference between fast and slow signal rates making the system resemble a one-pool system, while in juniper the compartments appear more separated. This indicates that the cycling of water between the xylem and the phloem occurs at different scales in pinon and juniper, and that the hydraulic conductivity between xylem and phloem and bark tissues could be higher in pinon than in juniper. Alternatively, even if the hydraulic conductivity was similar in these species, stronger water cycling between xylem and phloem could be obtained by higher osmotic concentrations in the phloem and bark tissue (Savage et al., 2015). The high rate of water cycling between the xylem and the phloem could explain the slow convergence of the signal in pinon. The NMR signal saturating, while the neutron signal did not, further confirms this interpretation. The majority of the observed NMR signal in our system seems to have been due to the water being rapidly transported through the xylem, while the neutron signal, linearly proportional to sample thickness, was more sensitive to the more stationary hydrogen reserves in the water in living cells and bark tissue.

## 4. Conclusions

We have demonstrated an ability to characterize changes in the water status of a tree over long time periods. This is done by removing the temperature dependence of NMR signals to isolate the water content. Large variations in water content indicate ready access to water; a constant water content indicates a lack of access to water; and a declining signal indicates drought or even tree death. Neutron imaging confirms that our water content measurement is primarily due to the water being rapidly transported through the plant. The neutron imaging experiments also showed how to observe water transport provided a suitable contrast agent, such as heavy water, can be introduced to the plant. While neutron imaging is a powerful and very sensitive tool for looking at plant water use, it is not a feasible approach for many experiments. In contrast our NMR system, while not as sensitive, is a noninvasive, scalable technique that can be fielded on many trees at once and outside of a strictly temperature controlled environment. Future work includes improving our ability to predict the NMR signal with temperature by modeling the flow of heat through the sample volume. Finally, a better understanding of the physical processes responsible for the variation in the water content could provide a tool to separate a tree's cavitation capacitance from its elastic capacitance.

## Author contributions

The concept of the NMR method and instruments was elaborated by SS, ME, MM, and JY. The NMR apparatus was designed and built by JY, HS, and MM. NMR data was acquired by JY and MM. NMR data analysis and theoretical work was by MM. The neutron imaging method and instruments were elaborated by RN, SV, and JH. Neutron imaging data was acquired by JH. Neutron imaging analysis was by JH, SS, and LD. MM and SS drafted the manuscript with critical input from all authors.

### Conflict of interest statement

The authors declare that the research was conducted in the absence of any commercial or financial relationships that could be construed as a potential conflict of interest.
